# Supplementary material for: ABF1 Positively Regulates Rice Chilling Tolerance via Inducing Trehalose Biosynthesis
Source: Int J Mol Sci. 2023 Jul 4;24(13):11082. doi: 10.3390/ijms241311082 (PMC10342119; doi:10.3390/ijms241311082)
Supplement: Supplementary file 1 [file ijms-24-11082-s001.zip › Supplemental Figures.pdf]

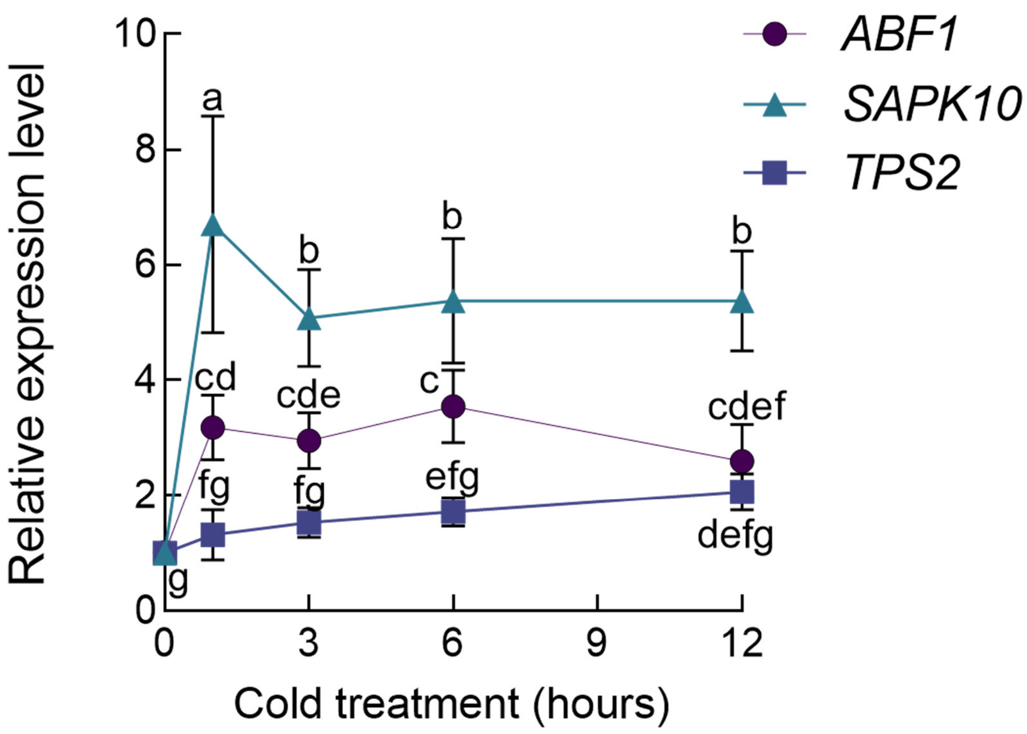

Figure S1. qRT-PCR analysis of *ABF1*, *SAPK10* and *TPS2* in seedling leaves of the wild type under chilling stress (4 °C). Error bars indicate SD with biological triplicates (n = 3). Tukey's test with two-way analysis of variance (ANOVA). Different letters indicate statistical differences at $p < 0.05$.

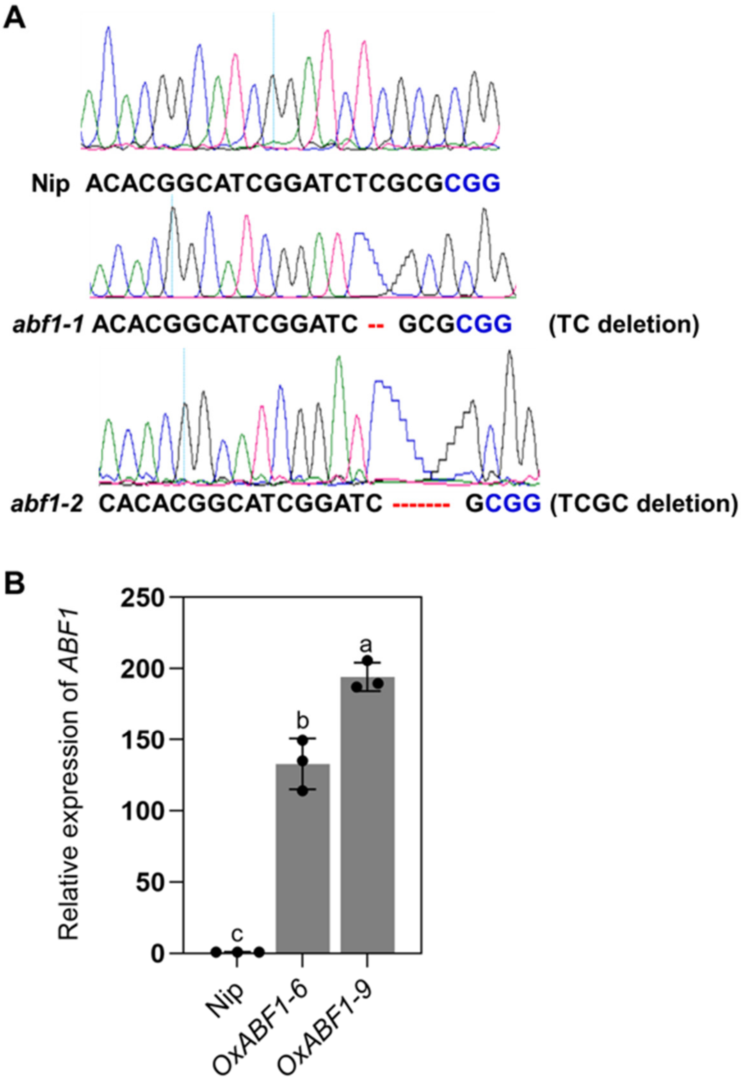

Figure S2. Molecular characterization of *abf1* mutant lines and *ABF1* overexpression lines. (A) Sanger sequencing chromatograph of the CRISPR-cas9 target site in homozygous mutant lines of *abf1* mutant lines. The letter in blue represented PAM. PAM: protospacer adjacent motif. (B) qRT-PCR of the transcription of *ABF1* in the seedlings of *ABF1* overexpression lines. Error bars indicate SD with biological triplicates (n = 3). Tukey's test with one-way analysis of variance (ANOVA). Different letters indicate statistical differences at $p < 0.05$.

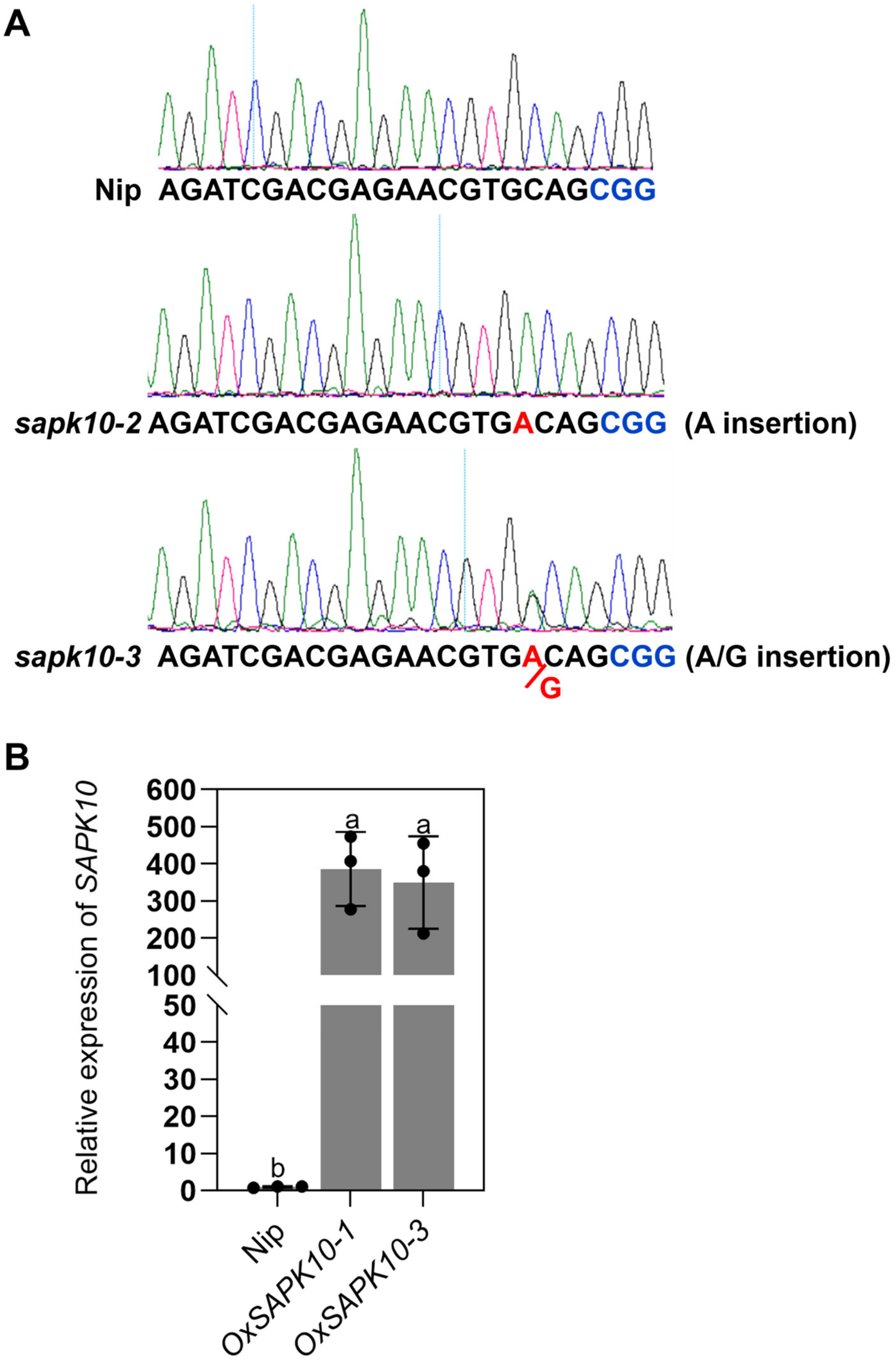

Figure S3. Molecular characterization of *sapk10* mutant lines and *SAPK10* overexpression lines. (A) Sanger sequencing chromatograph of the CRISPR-cas9 target site in homozygous mutant lines of *sapk10* mutant lines. The letter in blue represented PAM. PAM: protospacer adjacent motif. (B) qRT-PCR of the transcription of *SAPK10* in the seedlings of *SAPK10* overexpression lines. Error bars indicate SD with biological triplicates (n = 3). Tukey's test with one-way analysis of variance (ANOVA). Different letters indicate statistical differences at $p < 0.05$.

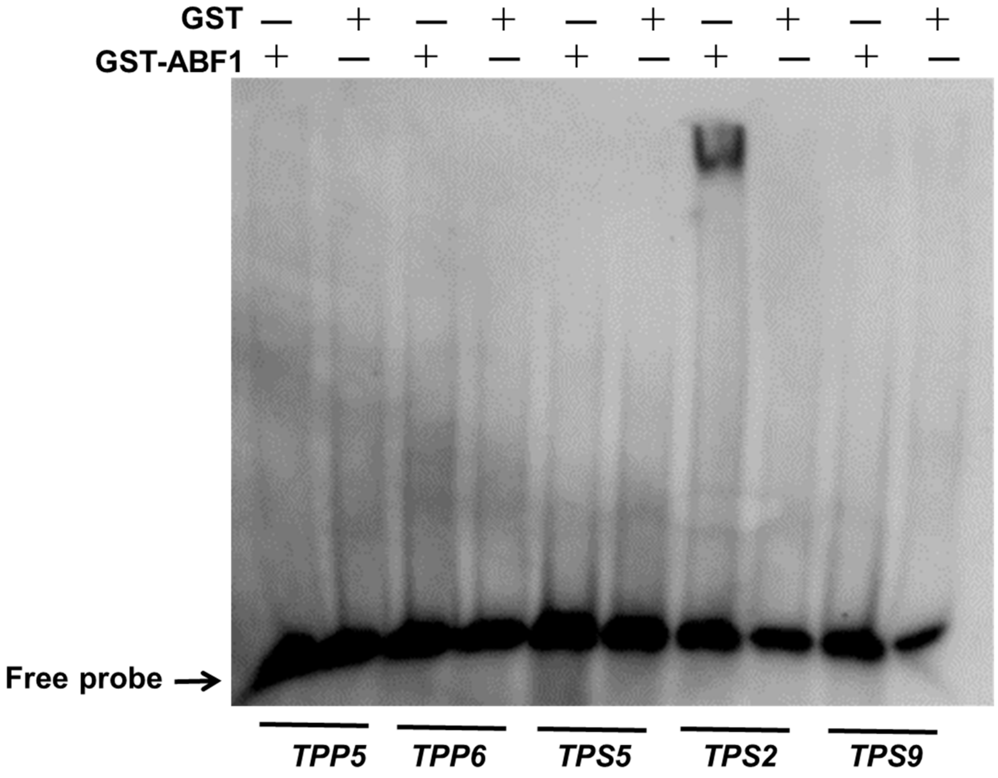

Figure S4. EMSA of ABF1 on trehalose biosynthesis pathway genes *TPP5, TPP6, TPS2, TPS5* and *TPS9* promoter regions.

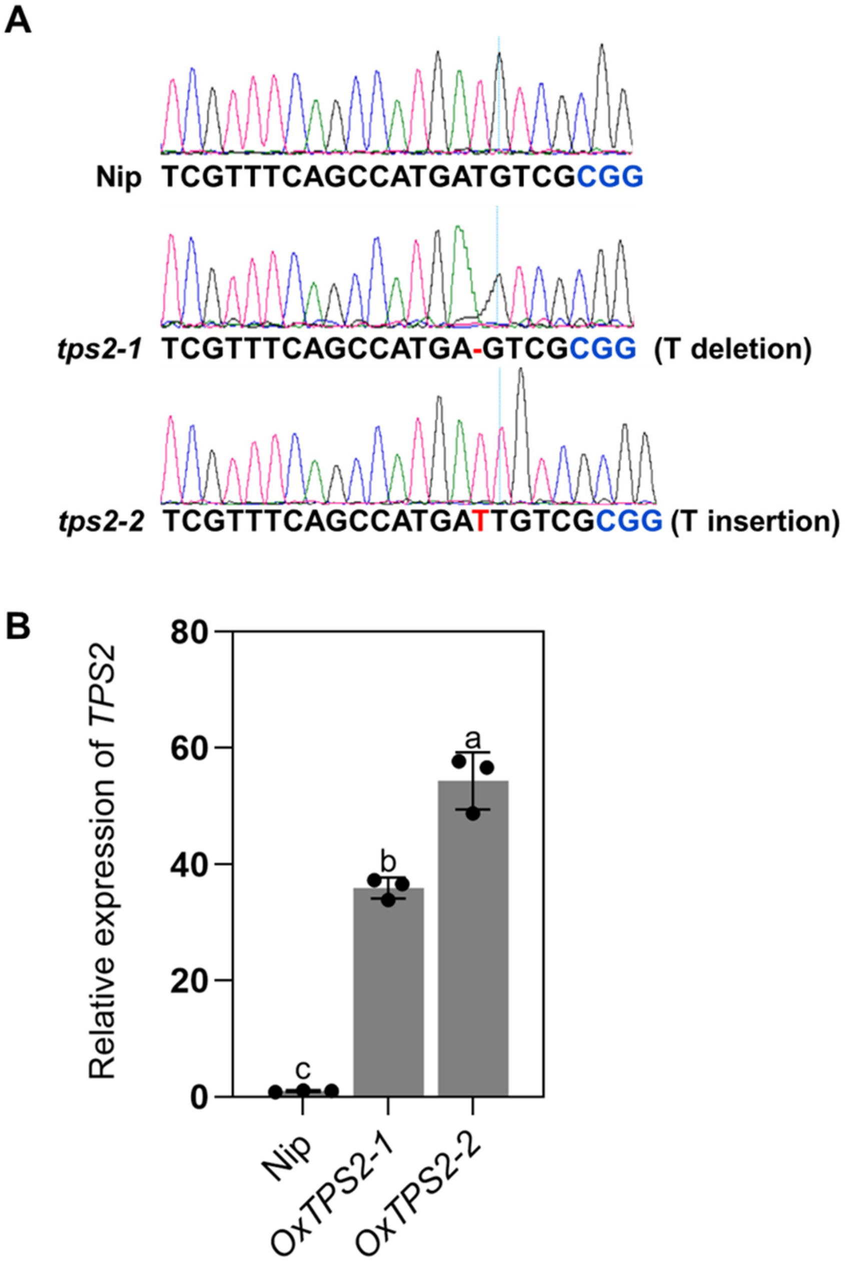

Figure S5. Molecular characterization of *tps2* mutant lines and *TPS2* overexpression lines. (A) Sanger sequencing chromatograph of the CRISPR-cas9 target site in homozygous mutant lines of *tps2* mutant lines. The letter in blue represented PAM. PAM: protospacer adjacent motif. (B) qRT-PCR of the transcription of *TPS2* in the seedlings of *TPS2* overexpression lines. Error bars indicate SD with biological triplicates (n = 3). Tukey's test with one-way analysis of variance (ANOVA). Different letters indicate statistical differences at $p < 0.05$.

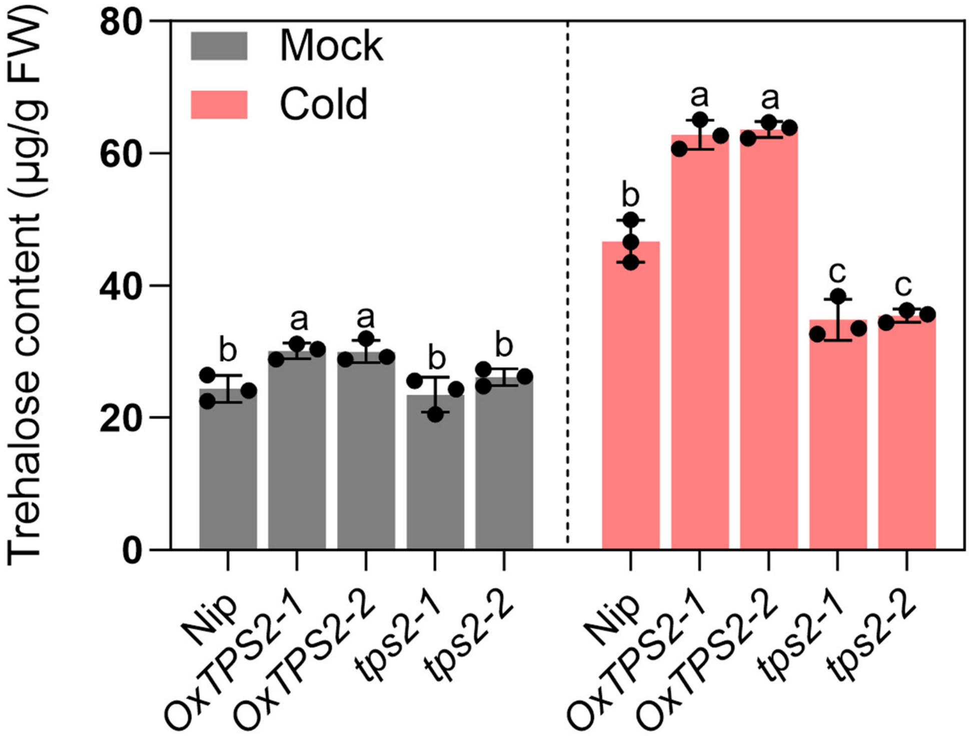

Figure S6. Trehalose contents of seedling leaves from wild type, tps2 *mutant lines* and TPS2 overexpression lines under chilling stress (4 °C) for 5 days. Error bars indicate SD with biological triplicates (n = 3). Tukey's test with one-way analysis of variance (ANOVA). Different letters indicate statistical differences at $p < 0.05$.
